# Social Stigmatization and Late Treatment of Dissociative Disorder: A Case Report on Trance and Possession Disorder

**DOI:** 10.7759/cureus.50198

**Published:** 2023-12-08

**Authors:** Imyarila Longkumer, Ragini Patil, Nayan Sinha, Namita Sahu

**Affiliations:** 1 Psychiatry, Jawaharlal Nehru Medical College, Datta Meghe Institute of Higher Education and Research, Wardha, IND

**Keywords:** mind body relation, shared psychosis, stigma, trance and possession, dissociative

## Abstract

Dissociative disorders have been present in our society since time immemorial, with culture, society, and spiritual beliefs playing a major part. It went through many changes from its name and what it constituted. Diagnosis can be made based on the International Classification of Diseases (ICD) 10, Diagnostic and Statistical Manual of Mental Disorders (DSM) 5, and now the current International Classification of Diseases (ICD) 11 criteria. One of its constituents is trance and possession disorder which is one of the main obstacles faced by psychiatrists in Indian society. Through this case, we can assume that there are many factors involved that lead to delays in diagnosing and managing a case of dissociative disorder, most importantly the social and cultural factors. Even now cultural-socio-spiritual beliefs add to the challenge of dissociative disorders. This case reveals that individuals first consult a general physician before visiting a psychiatrist; so, the need for consultation liaison psychiatry (CLP) and its role today in diagnosis and further management is emphasized. There still exists a need for awareness to be made regarding the mind-body relationship and psychosocial support to both patients and their relatives keeping in mind their beliefs.

## Introduction

The earliest case reports of dissociative disorder appeared at the end of the 18th century, and detailed descriptions of the condition first appeared in medical literature in the 19th century. In the 18th century, there was discussion on whether hysteria (somatoform symptoms) or dual consciousness (dissociation) should be used to categorize the condition. Somatoform hysteria eventually emerged as the conceptual basis for all of these illnesses [1]. A break in the normally integrated functioning of consciousness, memory, identity, or perception of the environment is known as dissociation [2]. Dissociation may be abrupt, acute, or insidious in onset. There are many types of dissociative disorder as per the International Classification of Disease (ICD) 10, namely dissociative amnesia, dissociative fugue, dissociative stupor, trance and possession disorders, dissociative motor disorders, dissociative convulsions, dissociative anesthesia, and sensory loss. The term "hysteria" has been used to describe these conditions in the past, but it currently appears preferable to avoid it as much as possible due to its many, and different connotations [3]. As per the International Classification of Disease (ICD) 10, trance and possession disorders are defined as states involving a temporary loss of the sense of personal identity and full awareness of the surroundings. The primary issue is the cross-cultural diversity of dissociative trance and possession states, like many mental conditions. Trance states exhibit variety in frequency and attribution, and they are perceived as manufactured in some cultures while pathological or socially acceptable in others. Behavior in those in possession and trance is frequently interpreted as being identical to changing that person's identity to that of another. Affected people may speak with different dialects or vocal tones or behave differently from other people [4]. The majority of people with dissociative disorders describe self-harming; up to 86% of dissociative people report having engaged in non-suicidal self-harm (NSSI) in the past, and up to 72% had attempted suicide at some point in their lives [5]. In a study, women with dissociative disorder in Magnetic Resonance Imaging (MRI) research showed considerably smaller hippocampus and amygdala sizes than healthy control subjects [6]. This case will bring out the multi-factorial reasons as to how dissociative disorder is a challenge to the psychiatrist and how it burdens the patient and their family.

## Case presentation

A 45-year-old married man from a low-income background came to the emergency room with a five-year history of limb weakness, unconsciousness, jerky movements, and memory problems after each episode. No prior psychiatric history in him or his family. He was moved to the intensive care unit (ICU) and assessed by a neurologist, with a normal electroencephalogram (EEG) result. He was then referred to psychiatry to investigate psychogenic nonepileptic seizure (PNES). Upon further evaluation, it was discovered that the family had concealed important details about his symptoms due to the stigma associated with mental disorders. The patient faced significant stressors, including strained relationships with his brother and son, as well as financial difficulties.

The patient had recurring 15-minute episodes marked by jerky movements, teeth clenching, and upward eye rolling, with no memory of them. Despite seeing multiple doctors and taking anticonvulsants, the episodes didn't improve; in fact, they became more frequent and longer. That same year, the family consulted a faith healer who alleged that another faith healer named "Narayan" was responsible for these symptoms as an act of revenge. Shortly after, the patient experienced another episode, exhibiting peculiar behavior. He believed he was under "Narayan's" influence, with an altered voice and distorted facial expressions. During these episodes, he attempted to harm himself and his family, with no recollection afterward.

He experienced episodes embodying an elderly neighbor and an unknown young girl, accompanied by second-degree auditory hallucinations where he heard these personas call him by his real name. This occurred while he was under a neurologist's care. Over five years, additional anticonvulsants like phenobarbitone and lacosamide were prescribed. The son noted that his mother and younger brother briefly encountered similar auditory hallucinations, with the young girl claiming to be sent by the faith healer "Narayan" for revenge, lasting only three days with no intervention. The family concealed both the stressor and these episodes from the doctor due to the stigma associated with them, resorting to multiple faith healers who reinforced their belief in possession and the voices.

The patient underwent a plain computed tomography (CT) brain and MRI brain, and blood tests, including a complete blood count, kidney function, and liver function, all showed results within the normal range. Consequently, the patient received a diagnosis of trance and possession disorder. Treatment began with 20 mg of fluoxetine capsules and 1000 mg of sodium valproate, which was gradually reduced and eventually discontinued.

The family and patient received psychoeducation about the disorder, how to recognize the episodes, and the importance of managing the behavior and committing to long-term treatment and follow-ups. The patient also received education on identifying triggers and learned breathing exercises and distraction techniques. Currently, after six months of follow-up, despite ongoing stressors regarding his strained relationship with his brothers and son, there have been no further episodes of trance and possession.

## Discussion

Dissociation/dissociative disorder diagnosis and treatment are a significant public health concern. Patients with dissociative disorder make up a sizable underserved community whose lack of acknowledgment results in high costs to individuals and society. Particularly males with dissociative disorder may go unnoticed because they are more likely to deny symptoms and traumatic history. In order to reduce the risk of suicide in both general and clinical populations, it is important to consider the strong link between dissociation, dissociative disorder, and suicidal and self-destructive behavior. The cultural and professional ignorance about the scope and gravity of the sort of trauma that causes dissociative disorder, as well as the prevalence of dissociative disorder patients, constitute the socio-cognitive issue. The human cost of improper diagnosis and treatment is quite substantial [7].

Disorders of dissociative trance and possession pose a challenge to psychiatry [8]. It is understood that culture influences the belief in the bounded individual self and depends on other cultural presumptions, which in turn affect these states [9]. Through the study of discrete behavioral states (DBS), dissociation research may also significantly advance our knowledge of the links between the mind, the brain, and the body. Many mind/brain/body mysteries in neuroscience, psychology, and psychiatry may be clarified through DBS models [10]. The clinician's job is to provide an alternate explanation for psychological conflicts and unmet needs, not to dismiss spiritual causes for reported symptoms [11].

Dissociative disorders may be triggered by co-occurring psychosis, affective illness, conflicts, or personality traits that reduce the person's capacity for adaptation, or more directly, they may cause the conscious or inadvertent use of dissociation to vent discontent and misery. Dissociation can be understood as a trauma response, as a way to convey emphatically how one feels out of control, confused, and overpowered., and as a tool to investigate and articulate one's position on more significant social, moral, and spiritual concerns [12]. The cornerstone of consultation liaison with psychiatry (CLP) is the integration of all information from all sources to deliver the best possible medical care. The care given should be sensible financially, sensitive to the needs of patients and other specialists, and attentive to prevention [13].

In the above case, delayed seeking of treatment secondary to stigma led to the worsening of the patient's symptoms. The culture and beliefs of the family also played a role in it as well. We see that dissociative disorder not only impacts the patient but also the family's mental health as in this case where his wife and son also had a brief period of secondary auditory hallucinations which resolved without any intervention. Many a time the physician may also dismiss patients’ beliefs affecting access to care leading to more increase in the illness burden on the patient and family. It also shows the importance of CLP for better patient management.

## Conclusions

Diagnosing and treating dissociative disorder is still a major challenge faced by the majority of psychiatrists. Even after that, educating the patient and relatives and how they perceive this illness also plays a role in the management while keeping in mind not to go against their belief. Adding to this challenge, the social and cultural barriers that come with it also add to its burden. There is a common belief even now that it is more predominant in females. Men with dissociative disorder and their families are more hesitant to visit doctors due to them playing the dominant masculine gender roles, making it more difficult to treat them. Delay in treatment also may have led to shared psychosis for a brief period which couldn’t be assessed in detail here. Consultation liaison with psychiatry also plays a very important role in diagnosing and managing many psychiatric illnesses including dissociative disorder as seen in this case.
